# Homœopathic Dilutions

**Published:** 1892-02

**Authors:** S. Mills Fowler

**Affiliations:** Gainesville, Texas


### HOMŒOPATHIC DILUTIONS.

EDITOR OF THE HOMŒOPATHIC PHYSICIAN:

I have been waiting for time, and "the spirit to move me" to say something regarding your editorial in the October number of THE HOMŒOPATHIC PHYSICIAN, at page 377.

In all of the universe of "matter" and "force" there is nothing more strange, mysterious, or inexplicable than the operation of "*homœopathic*" remedies.

It seems to me that your explanation partakes too strongly of the "*material*."

It seems to me, further, that any explanation that we may offer from a materialistic stand-point, is far from satisfying. Any explanation, in fact, that can be offered is only speculative.

Disease is not an entity. What better can we call it than "*perverted energy*"? The varying phases of disease are owing to idiosyncrasy, predisposing and exciting causes.

Homœopathic remedies are not entities, but are the different forms or modes of energy inherent in substance.

By the processes of trituration, succussion, etc., these different forms of energy are transferred to inert media, and there held in suspension, ready for use. There is no substance but is possessed of some form of energy, when subjected to those conditions which call it into action.

Drug, or remedial energy, like disease energy, belongs to the realm of metaphysics; and can only be studied by their metaphysical relation to each other, and by their effects, and not by their physical properties, or any natural (as popularly understood) phenomena; and are not amenable to the laws governing material things.

The force inherent in the Natrum-muriaticum crystals is that peculiar energy which makes it Natrum-muriaticum and not Natrum-sulphuricum, or any other substance. By triturating those crystals with sugar of milk, the *material* of the salt is dissipated, and its peculiar energy transferred to the sugar of milk. This process of trituration, succussion, etc., does not, as some claim, convert the whole mass into a *dilution* of Natrum-muriaticum, but gives the *impulse* which transforms the whole into a *potentization* of Natrum-muriaticum, possessing peculiar but wonderful properties, differing widely from the original crude material.

Given a case of "perverted energy" (sickness), the symptoms of which call for the exhibition of Natrum-muriaticum (or any remedy). *We cannot explain* HOW the remedial energy operates to cause a suspension of those symptoms, or a return to healthfulness, any more than we can explain how the apple falls, or the grass grows; that is where the mystery lies. All we can say is that it is a manifestation of the operation of remedial energy, in accordance with the law of similars; and all that we can know about it is that the effects are sure and unvarying.

It is the energy, liberated as it were from its prison in the crystal that is operative in the potentized (dynamized) preparation of the Natrum-muriaticum (or any other substance), and shows its effect when brought into company—so to speak—with its nearly-related similar disease energy, and explains why it is comparatively inert in the substance, or crude material.

"Matter is made up of atoms," as you say. But matter as such is void of force or energy, and it is only after all of the physical characteristics of matter have been destroyed, far beyond the reach of the most delicate tests of science to detect, that we obtain that energy which is capable of moving in that higher realm of life where disease energy is manifested.

S. MILLS FOWLER, M. D.

GAINESVILLE, TEXAS, Dec. 25th, 1891.

---

